# Supplementary material for: Three-dimensional domain identification in a single hexagonal manganite nanocrystal
Source: Nat Commun. 2024 Apr 27;15:3587. doi: 10.1038/s41467-024-48002-z (PMC11055849; doi:10.1038/s41467-024-48002-z)
Supplement: Supplementary file 1 — Supplementary Information [file 41467_2024_48002_MOESM1_ESM.pdf]

# Supplementary Material for: Three-Dimensional Domain Identification in a Single Hexagonal Manganite Nanocrystal

**Ahmed H. Mokhtar[1*], David Serban[1], Dan Porter[3], Frank Lichtenberg[2], Stephen P. Collins[3], Alessandro Bombardi[3], Nicola Spaldin[2], Marcus C. Newton[1*]**

[1] School of Physics and Astronomy, University of Southampton. University Road, Southampton, UK, SO17 1BJ
[2] Department of Materials, ETH Zurich. Ramistrasse 101, 8092 Zurich, Switzerland
[3] Beamline I16, Diamond Light Source. Harwell Science and Innovation Campus, Didcot, Oxfordshire, UK, OX11 0DE

---

**Supplementary Note 1**

The following describes how to calculate the $Q$-vector for a given diffractometer geometry. We will consider a 4S+2D diffractometer using two differing (rectilinear) coordinate systems.

The general (right-handed) rotation matrices about each axis, independent of the orientation of a chosen (rectilinear) coordinate system are:

$$R_{\hat{x}}(\theta) = \begin{bmatrix} 1 & 0 & 0 \\ 0 & \cos\theta & -\sin\theta \\ 0 & \sin\theta & \cos\theta \end{bmatrix} \tag{1}$$

$$R_{\hat{y}}(\theta) = \begin{bmatrix} \cos\theta & 0 & \sin\theta \\ 0 & 1 & 0 \\ -\sin\theta & 0 & \cos\theta \end{bmatrix} \tag{2}$$

$$R_{\hat{z}}(\theta) = \begin{bmatrix} \cos\theta & -\sin\theta & 0 \\ \sin\theta & \cos\theta & 0 \\ 0 & 0 & 1 \end{bmatrix} \tag{3}$$

The coordinate system assumes the beam oriented along the positive $\hat{y}$ direction, the $\hat{x}$ direction pointing upwards and $\hat{z}$ direction along the $\hat{x} \times \hat{y}$ direction [1, 2]. Rotations about $\mu$, $\eta$, $\chi$ and $\phi$ according this coordinate system are as follows:

$$\begin{aligned} R_{\mu}(\mu) &= R_{\hat{x}}(\mu) \\ R_{\eta}(\eta) &= R_{\hat{z}}(-\eta) \quad \text{i.e. left handed} \\ R_{\chi}(\chi) &= R_{\hat{y}}(\chi) \\ R_{\phi}(\phi) &= R_{\hat{z}}(-\phi) \quad \text{i.e. left handed} \\ R_{\Delta}(\delta) &= R_{\hat{z}}(-\delta) \quad \text{i.e. left handed} \end{aligned} \tag{4}$$

To obtain the complete rotation matrix, matrices in Equation 4 are multiplied form the outer most circle ($\mu$) to the inner most circle ($\phi$). Noting that the X-ray beam ($\mathbf{k}_i$) is aligned along the positive $\hat{y}$ direction with magnitude $k$,

$$\mathbf{k}_i = \begin{bmatrix} 0 \\ k \\ 0 \end{bmatrix} \tag{5}$$

the reflected wavevector ($\mathbf{k}_f$) is given by:

$$\mathbf{k}_f = \begin{bmatrix} k_{f\hat{x}} \\ k_{f\hat{y}} \\ k_{f\hat{z}} \end{bmatrix} = R_\Delta \mathbf{k}_i \tag{6}$$

Using Equations 5 and 6, the $\mathbf{Q}$-vector is then obtained in the laboratory frame of reference as:

$$\mathbf{Q} = \mathbf{k}_f - \mathbf{k}_i \tag{7}$$

The standard coordinate transform in Bonsu [3], however uses a standard 4S+2D diffractometer right handed coordinate system where the X-ray beam is assumed to align along the positive $\hat{z}$ direction, the $\hat{x}$ direction pointing upwards as before, but the $\hat{y}$ direction pointing along the $\hat{z} \times \hat{x}$ direction.

In this system, rotations about $\mu$, $\eta$, $\chi$ and $\phi$ are as follows:

$$\begin{aligned} R_\mu(\mu) &= R_{\hat{x}}(\mu) \\ R_\eta(\eta) &= R_{\hat{y}}(\eta) \\ R_\chi(\chi) &= R_{\hat{z}}(\chi) \\ R_\phi(\phi) &= R_{\hat{y}}(\phi) \\ R_\Delta(\delta) &= R_{\hat{y}}(\delta) \end{aligned} \tag{8}$$

The X-ray beam ($\mathbf{k}_i$) is aligned along the positive $\hat{z}$ direction with magnitude $k$,

$$\mathbf{k}_i = \begin{bmatrix} 0 \\ 0 \\ k \end{bmatrix} \tag{9}$$

where $k = \frac{4\pi sin(\theta)}{\lambda}$ is the wave vector magnitude.The $\mathbf{Q}$-vector is then obtained in the laboratory frame of reference as before, as shown above.

To correct multi-Bragg rotations, each $\mathbf{Q_n}$-vector obtained using the above methods **and** each coordinate corrected diffraction pattern, is rotated back to a position $\mathbf{Q}_n^*$ where the $\mathbf{k}_f$ vector aligns parallel to the $\mathbf{k}_i$ vector. This ensures that the angles between each $\mathbf{Q}$-vector correspond to the correct direction in reciprocal space:

$$\mathbf{Q}_n^* = R_\phi(-\phi)R_\chi(-\chi)R_\eta(-\eta)R_\mu(-\mu)\mathbf{Q}_n \tag{10}$$

$$\rho_n^*(\mathbf{q}) = R_\phi(-\phi)R_\chi(-\chi)R_\eta(-\eta)R_\mu(-\mu)\rho_n(\mathbf{q}) \tag{11}$$

An alternative approach is to rotate all vectors relative to a choice of $\mathbf{Q_n}$-vector. This produces equivalent results.

**Supplementary Note 2**

| Reflection | $\chi$ | $\eta$ | $\phi$ |
|---|---|---|---|
| 110 | 66.63 | 9.105 | 33.476 |
| 111 | 80.22 | 2.947 | 44.98 |
| 11m1 | 53.33 | 16.39 | 24.81 |
| 212 | 76.59 | 1.69 | 31.50 |
| 300 | 45.29 | 6.31 | 0.488 |

**Supplementary Table 1.** The diffractometer rotations done to align each of the reflections to $\mathbf{Q_n} = \mathbf{G}$.

| Reflection | $Q_x$ | $Q_y$ | $Q_z$ |
|---|---|---|---|
| 110 | 16.67 | -41.75 | 7.49 |
| 111 | 11.16 | -44.19 | -0.39 |
| $11\bar{1}$ | 23.63 | -36.50 | 13.65 |
| 212 | 16.42 | -41.81 | -7.732 |
| 300 | 30.87 | -31.07 | -12.61 |

**Supplementary Table 2.** The components of the calculated Q vectors after geometry corrections for each of the 5 reflections.

**Supplementary Note 3**

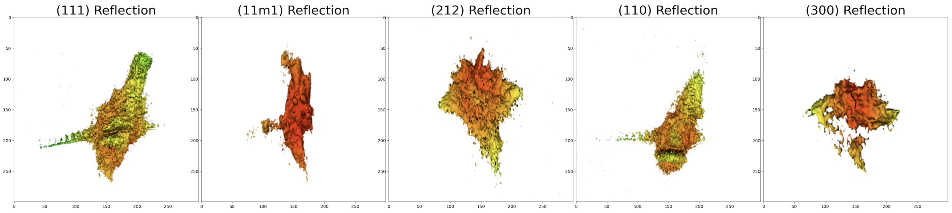


**Supplementary Figure 1.** 3-dimensional rendering of the collected diffraction patterns. The colour map represents the amplitude.

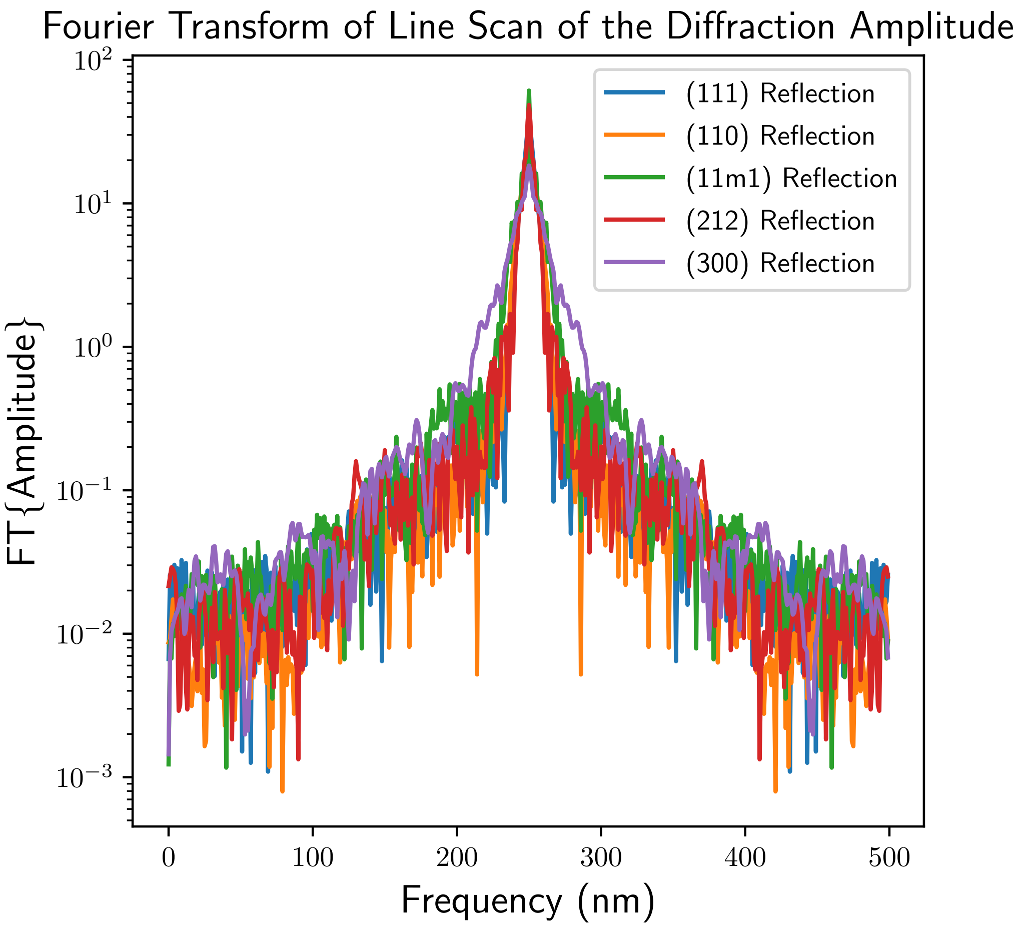


**Supplementary Figure 2.** The Fourier transform of line scans through each of the diffraction patterns which illustrates the consistency in the frequency of fringes.

## Supplementary Note 4

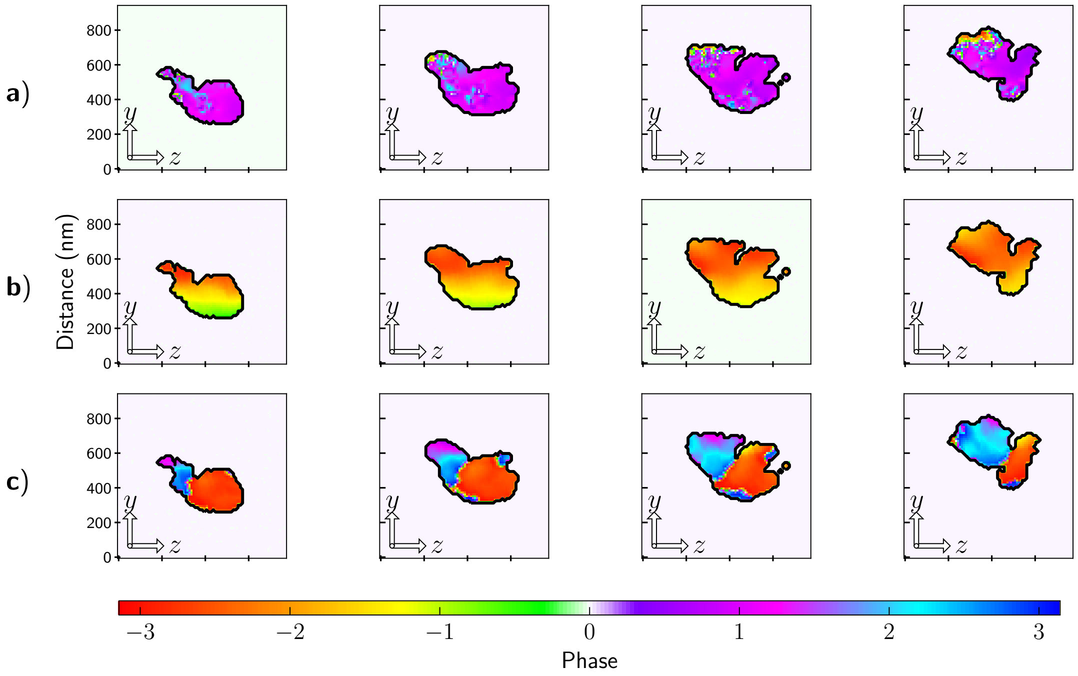


**Supplementary Figure 3.** Cross-Sectional planes of the phase maps of the (a)(111) (b) (110) and (c)(212) reflections at different points in the reconstructed crystal.

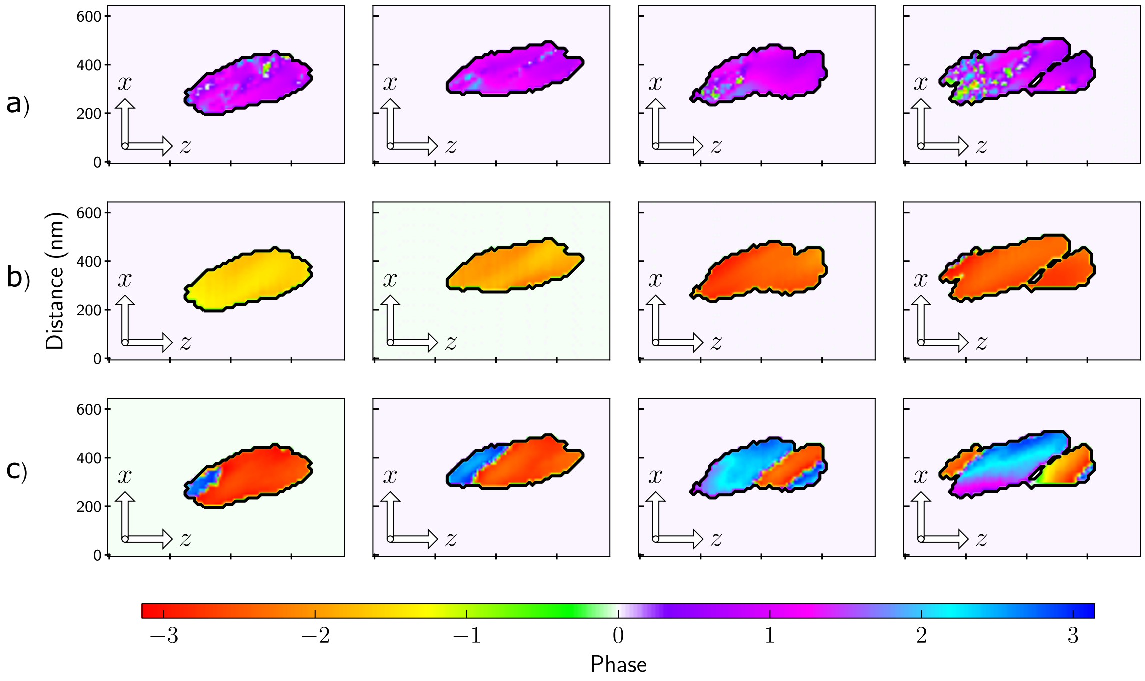


**Supplementary Figure 4.** Cross-Sectional planes of the phase maps of the (a) (111) (b) (110) and (c) (212) reflections at different points in the reconstructed crystal.

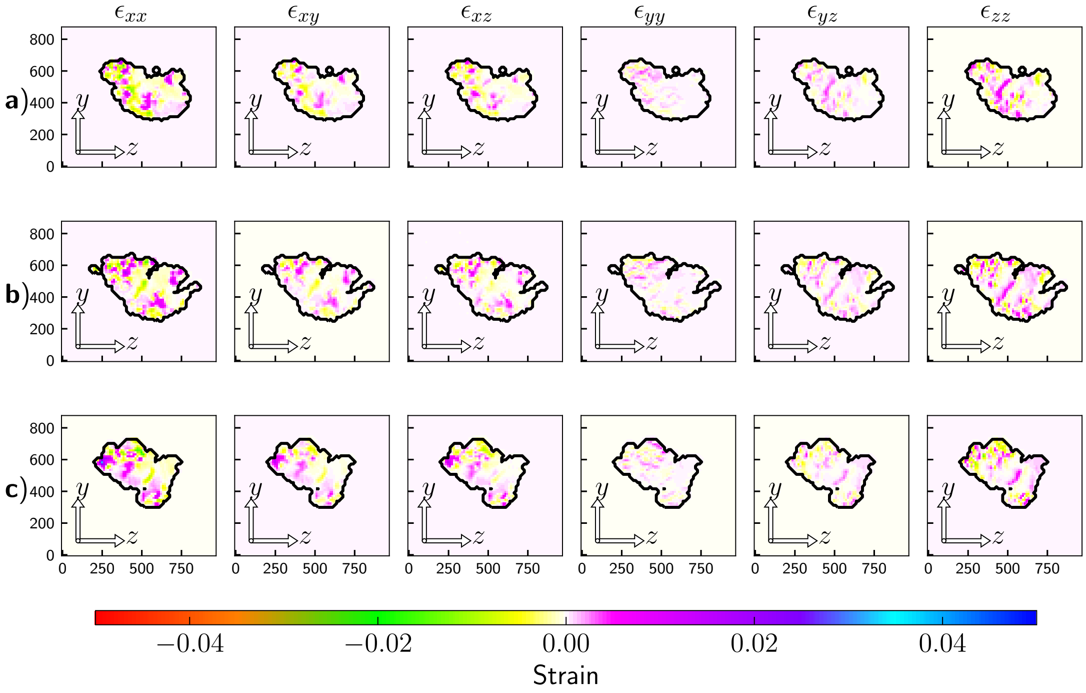


**Supplementary Figure 5.** Strain tensor maps for all components perpendicular to the x-axis direction taken at the a) first, b) section and c) third locations the phase maps.

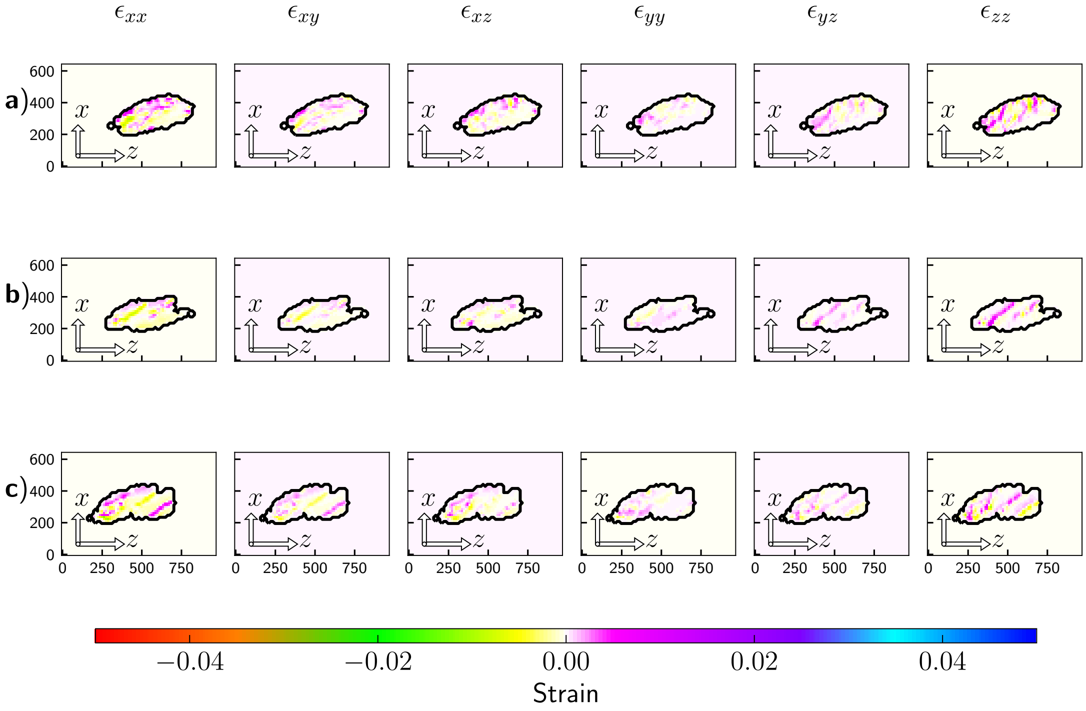


**Supplementary Figure 6.** Strain tensor maps for all components perpendicular to the y-axis direction taken at the a) first, b) section and c) third locations the phase maps.

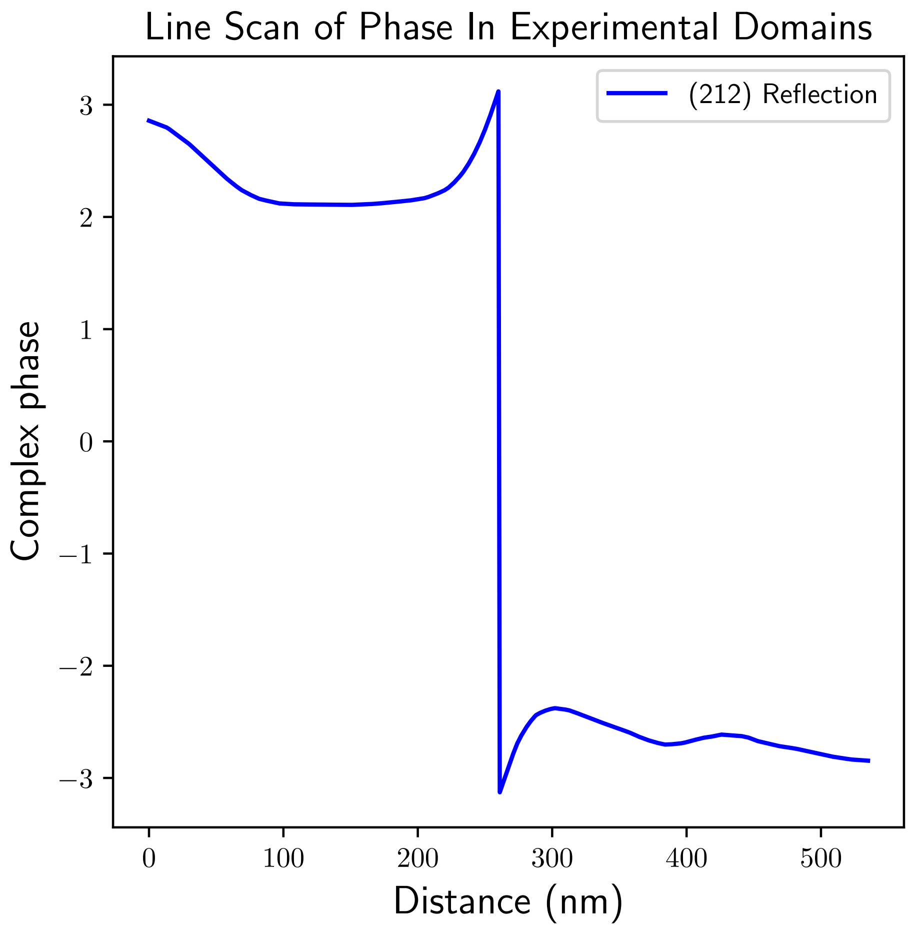


**Supplementary Figure 7.** A line scan of the reconstructed phase for the (212) reflection across the two domains. We can observe the nearly constant phase and the symmetrical nature of the phase across the two domains.

**Supplementary Note 5**

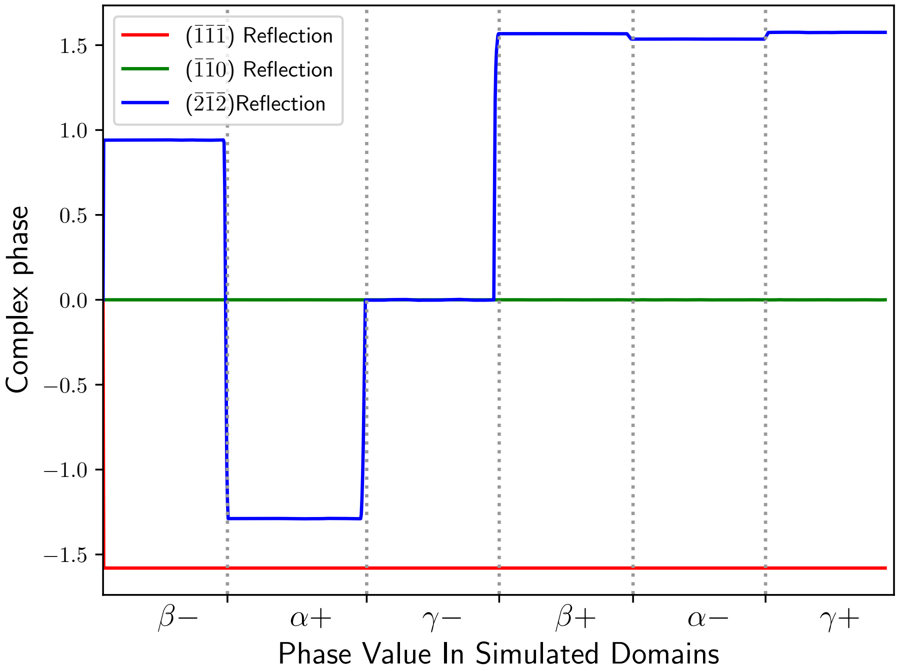


**Supplementary Figure 8.** A line scan of the simulated phase for the -**Q** of the three reflections. We observe the phase values of each -**Q** to be the exact negative of the +**Q**.

By examining the reconstructed phase in conjunction with the relative angles between the scattering vectors corresponding, we utilise reconstructed the phase information as an additional layer of discrimination between +**Q** and −**Q**, as the crystal structure is not centrosymmetric. Specifically, the simulated data for the (111) reflection indicates a positive phase, contrasting with a negative phase for the $(\bar{1}\bar{1}\bar{1})$ reflection. This phase relationship suggests that the experimental (111) reflection corresponds to a positive **Q** direction as it also demonstrated positive phase, leading to the correct identification of the +**Q** rather than its Friedal pair for the experimental (111) reflection.

The geometrical relationships between between pairs of **Q**-vectors can be analysed to determine whether we are observing +**Q** or -**Q**. For instance, the angle between (111) and $(\bar{2}\bar{1}\bar{2})$ reflections is significantly different from that between (111) and (212), with measured angles of 168.7° and 11.28°, respectively. Our experimental value of the relative angles between these Q vector matches the latter, validating the direction of the (212) to be the +Q direction. This in turn validates the choice for the simulation, and validates that we have the correct polarity for the experimental circular mean.

An additional layer of evidence comes from the necessity for consistency among all reflections during concurrent phase retrieval. This process inherently penalizes and filters out inconsistent reflections, which would not reconstruct accurately if the assumed **Q**-vector orientations were incorrect. The successful reconstruction of our dataset, with all reflections showing consistent phase relations as per our simulations, further validates our methodology and conclusions. These arguments, in conjunction with the matching magnitude of the circular mean of the (212) reflection with that of the simulated data, provide a robust argument supporting the identification of +**Q** vector orientations. There is no matching magnitude of the circular mean if we were observing -**Q**.

## References

[1] You, H. Angle calculations for a '4S+2D' six-circle diffractometer. *J. Appl. Crystallogr.* **32**, 614-623 (1999).

[2] Busing, W. & Levy, H. Angle calculations for 3- and 4-circle X-ray and neutron diffractometers. *Acta Crystallogr.* **22**, 457-464 (1967).

[3] Newton, M., Nishino, Y. & Robinson, I. Bonsu: the interactive phase retrieval suite. *J. Appl. Crystallogr.* **45**, 840-843 (2012).
