# Supplementary material for: Three-dimensional domain identification in a single hexagonal manganite nanocrystal
Source: Nat Commun. 2024 Apr 27;15:3587. doi: 10.1038/s41467-024-48002-z (PMC11055849; doi:10.1038/s41467-024-48002-z)
Supplement: Supplementary file 3 — Description of Additional Supplementary Files [file 41467_2024_48002_MOESM3_ESM.pdf]

Description of Additional Supplementary Files

Supplementary Movie 1: A 3D rendering of the reconstructed displacement field, as flow lines.
